# The Overlooked Biodiversity of Flower-Visiting Invertebrates

**DOI:** 10.1371/journal.pone.0045796

**Published:** 2012-09-19

**Authors:** Carl W. Wardhaugh, Nigel E. Stork, Will Edwards, Peter S. Grimbacher

**Affiliations:** 1 School of Marine and Tropical Biology, James Cook University, Smithfield, Queensland, Australia; 2 Griffith School of Environment, Griffith University, Nathan, Queensland, Australia; 3 Department of Resource Management and Geography, University of Melbourne, Richmond, Victoria, Australia; University of Northampton, United Kingdom

## Abstract

Estimates suggest that perhaps 40% of all invertebrate species are found in tropical rainforest canopies. Extrapolations of total diversity and food web analyses have been based almost exclusively on species inhabiting the foliage, under the assumption that foliage samples are representative of the entire canopy. We examined the validity of this assumption by comparing the density of invertebrates and the species richness of beetles across three canopy microhabitats (mature leaves, new leaves and flowers) on a one hectare plot in an Australian tropical rainforest. Specifically, we tested two hypotheses: 1) canopy invertebrate density and species richness are directly proportional to the amount of resource available; and 2) canopy microhabitats represent discrete resources that are utilised by their own specialised invertebrate communities. We show that flowers in the canopy support invertebrate densities that are ten to ten thousand times greater than on the nearby foliage when expressed on a per-unit resource biomass basis. Furthermore, species-level analyses of the beetle fauna revealed that flowers support a unique and remarkably rich fauna compared to foliage, with very little species overlap between microhabitats. We reject the hypothesis that the insect fauna on mature foliage is representative of the greater canopy community even though mature foliage comprises a very large proportion of canopy plant biomass. Although the significance of the evolutionary relationship between flowers and insects is well known with respect to plant reproduction, less is known about the importance of flowers as resources for tropical insects. Consequently, we suggest that this constitutes a more important piece of the ‘diversity jigsaw puzzle’ than has been previously recognised and could alter our understanding of the evolution of plant-herbivore interactions and food web dynamics, and provide a better foundation for accurately estimating global species richness.

## Introduction

Current estimates suggest that approximately 40% of all invertebrate species utilise rainforest canopies [1]. In these systems invertebrates typically represent the most diverse, abundant and effective pollinators [2], herbivores [3], and predators [4]. High species richness and functional diversity of canopy plants and animals and the relationships that develop between them are strongly influential in determining food web dynamics [5], and have been used to estimate global species richness [6]–[11].

While the high diversity of invertebrates in rainforest canopies has been recognized for some decades [6], [12]–[14], difficulties in accessing the canopy have limited many previous biodiversity and ecological studies to mass sampling techniques that indiscriminately sample many arboreal microhabitats together; such as insecticide fogging [6], [13], [14] or flight interception/Malaise traps [15]. Exceptions include sampling individual parts of trees using techniques such as enclosed gassing [16] and branch clipping [17], which provide more localised information on invertebrate communities. Although rainforest canopies contain a range of resources, such as leaves, flowers, fruits, bark, epiphytes, and living and dead wood that may be exploited by invertebrates, most studies that have used discrete sampling techniques were restricted to single microhabitat types [18]. In general, canopy invertebrate biodiversity studies have been largely restricted to mature foliage [18], since this represents the most abundant biomass in rainforest canopies. The practical result of this sample bias is that it remains unknown whether samples taken from mature foliage accurately reflect abundances and diversity in the canopy as a whole. Consequently, generalisations about distribution patterns and food web dynamics are difficult to make since we know very little about habitat differentiation in rainforest canopies, or how species are divided across microhabitats.

There is a *prima facie* reason to expect that samples from a single resource type such as leaves are unlikely to represent the diversity or composition of all possible resources in rainforest canopies. First, resource differentiation and niche-based theories predict specialisation on different microhabitats (e.g., [19]). For example, feeding trials have shown that many herbivores are restricted to feeding on new leaves, and are unable to consume fully expanded mature foliage [20] suggesting they will be underrepresented (or undetected) in samples taken from mature foliage. Second, the very small amount of empirical evidence available is strongly in favour of different assemblages associated with different resources. For example, 90/138 (65%) flower-feeding caterpillar species from Brazilian Cerrado were not recorded from foliage during 17 years of sampling [21], indicating that different host plant microhabitats are inhabited by discrete, largely non-overlapping invertebrate communities.

To date, few studies have simultaneously compared the invertebrate faunas of more than one microhabitat from tropical rainforest canopy trees. Significant differences in community composition have been found between the faunas inhabiting epiphyte associated habitats and host tree microhabitats [22], [23]. Ødegaard [24], [25] examined the host specificity of the foliage, flower, and dead wood-inhabiting herbivorous beetle (Buprestoidea, Chrysomeloidea, and Curculionoidea) communities in Panama. He showed that the flower-visitor assemblage was diverse (flower-visitors made up ∼20% of all beetle species collected), less host specific than folivores, and unique from the communities inhabiting the other focal microhabitats [24]. Furthermore, the beetle assemblage on suspended dead wood on one tree species, *Brosimum utile* (Moraceae), was complementary to that on the leaves, and even more diverse [25].

Results from studies in other fields also point to an expectation of differences in assemblage structure and diversity between microhabitats. For example, pollination studies have shown that flowers represent especially important sites of diversity [26]. Indeed, the evolution of insect pollination systems is thought to have been a major driver in the diversification of angiosperms [27], [28], and it is estimated that over 90% of tropical rainforest trees are pollinated by insects [2], [29], [30]. However, the hypothesis that angiosperm diversification was the result of specialist one-on-one pollination syndromes remains controversial, since plant species with generalised insect pollination systems that attract a suite of insect floral visitors outnumber specialist systems [2], [31]. Furthermore, numerous flower-visiting species are not actively involved in pollination [26], [32], but may be associated with flowers because they consume nectar, pollen [33], oils [34], floral parts [35], [36], or because they are predators of other flower-visitors [37], [38]. Flowers therefore, should be expected to support a large number of insect species. Unfortunately, difficulty accessing rainforest canopy flowers has meant that little collecting from this microhabitat has occurred, especially by those undertaking biodiversity studies, so this community has been largely ignored.

Understanding how biodiversity influences ecological processes requires a detailed understanding of how species are distributed [39]. Although some studies have examined microhabitat differentiation among tropical rainforest canopy invertebrate assemblages [22]–[25] none recorded the biomass of each microhabitat. The extent to which species richness and density vary between canopy microhabitats therefore remains unknown. This knowledge is required for detailed examinations of rainforest food webs and the strength and nature of intra- and interspecific interactions, which have important implications for the evolution of insects and their host plants.

Despite the obvious importance that understanding the distribution and diversity of invertebrates in canopies has for quantifying biodiversity and food web dynamics, few have quantified differences in tropical insect assemblages inhabiting multiple canopy microhabitats. Here, we compare the abundance, density per unit dry weight, species richness and compositional overlap of the invertebrate communities between canopy microhabitats. Specifically, we examine the invertebrate assemblages on mature leaves, new leaves, and flowers from 23 species of rainforest canopy plants to determine the relative contribution of each microhabitat to canopy invertebrate diversity. First we tested the null hypothesis that canopy invertebrate density and species richness are directly proportional to the amount of resource available. Second, we tested the hypothesis that canopy microhabitats represent discrete resources that are utilised by their own specialised invertebrate communities. This approach allowed for an assessment of the validity of using leaf-based samples to capture representative canopy-wide patterns in invertebrate abundance, density and species richness. We speculate as to why there are differences in the abundance and diversity of invertebrates between the sampled microhabitats. Elsewhere we have used these same samples to show that the composition [40], feeding guild structure [41] and host specificity (Wardhaugh et al. unpublished data) of the diverse beetle community varies substantially between assemblages collected from different canopy microhabitats.

## Methods

### Study site

All fieldwork was conducted using the Australian Canopy Crane (www.jcu.edu.au/canopycrane/) at the Daintree Rainforest Observatory (a Long-Term Ecological Research site), near Cape Tribulation (16°17′S, 145°29′E) Queensland, Australia [42]. The crane is situated approximately 40 m a.s.l. and >300 m from the forest edge in complex mesophyll vine forest [43] that is contiguous with the extensive lowland and upland rainforests of the Daintree National Park and Wet Tropics World Heritage Area (0 m a.s.l.–>1300 m a.s.l.). Approximately 1 ha of rainforest containing 745 individual trees (>10 cm d.b.h.) from 82 species and 34 families is accessible from the crane gondola (based on a recent (2009) survey at the crane site which updates previously published data [44]). The canopy is noticeably uneven in height, varying from 10 to 35 m. Although some rain does fall each month (the lowest average monthly rainfall occurs in August; 80 mm), there is a distinctive wet season from November–April (the highest average monthly rainfall occurs in March; 550 mm). The 50 year average annual precipitation at Cape Tribulation is 3996 mm [45].

### Sampling Methods

Invertebrates were sampled from three microhabitats; mature leaves, new leaves, and flowers, from 23 locally common canopy plant species. Fruit and suspended dead wood were also sampled but were scarce in the canopy and poorly utilised by externally feeding invertebrates. Consequently, fruit and dead wood invertebrate communities were omitted from the analyses presented here. Epiphytes are rare at the crane site and were not sampled for this reason. The host tree species selected represent a broad range of taxonomic relatedness, growth pattern, phenology, distribution, size, and abundance. In addition to woody trees (19 species), two species of palms and two species of lianas were sampled (Table 1). These species comprise 435/745 individuals and >70% of the basal area of all trees >10 cm d.b.h. in the ∼1 ha area of forest directly under the crane [44]. One to three individuals of each host species were sampled each month for one year (May 2008–May 2009). Sampling did not occur in October 2008 due to the temporary unavailability of the crane. Invertebrate sampling was carried out by hand collecting all observable individuals, as well as beating the microhabitat over a beating sheet to dislodge cryptic species. No sampling technique is truly representative and each suffers some degree of sampling bias. The hand collecting and beating techniques we used are generally biased towards taxonomic groups that are flightless or flight-reluctant, such as beetles, spiders and ants. Consequently, strong flying groups, such as flies and wasps, may be underrepresented in our samples. Only externally active invertebrates were collected, and no attempt was made to include species within plant tissue, such as leaf miners and gallers. Each microhabitat on each replicate tree was sampled for ten minutes. In general, trees that were flowering and/or leaf flushing were selected wherever possible, to maximise the number and temporal distribution of samples from these more ephemeral microhabitats. Cross contamination between microhabitat samples was kept to a minimum by only sampling microhabitats that were discretely partitioned on host trees.

**Table 1:** The canopy plant species sampled, including the number of trees on site, the number of trees sampled, and the number of mature leaf, new leaf, and flower samples from each tree species.

Habit	Family	Species	Trees on site	No. individuals sampled	No. mature leaf samples	No. new leaf samples	No. flower samples
Trees	Lauraceae	*Endiandra microneura*	22	3	20	4	0
		*Cryptocarya mackinnoniana*	16	4	14	6	0
		*Cryptocarya grandis*	7	2	1	2	2
		*Cryptocarya hypospodia*	1	1	3	0	1
	Myrtaceae	*Acmena graveolens*	16	6	19	5	5
		*Syzygium sayeri*	9	5	20	3	6
		*Syzygium gustavioides*	8	4	10	11	22
	Meliaceae	*Dysoxylum papuanum*	12	3	21	4	2
		*Dysoxylum pettigrewianum*	9	3	19	5	0
	Euphorbiaceae	*Cleistanthus myrianthus*	90	3	23	1	0
	Apocynaceae	*Alstonia scholaris*	61	4	20	3	0
	Elaeocarpaceae	*Elaeocarpus angustifolius*	7	4	22	0	2
		*Elaeocarpus bancrofti*	1	1	11	1	1
	Cunoniaceae	*Gillbeea whypallana*	5	1	3	3	2
	Proteaceae	*Cardwellia sublimis*	14	4	20	6	2
		*Musgravia heterophylla*	7	1	0	0	1
	Sterculiaceae	*Argyrodendron peralatum*	17	3	16	4	7
	Myristicaceae	*Myristica insipida*	59	3	18	2	3
	Fabaceae	*Castanospermum australe*	8	4	22	4	2
Lianas		*Entada phaseoloides*		5	17	7	2
	Convolvulaceae	*Merremia peltata*		9	19	3	5
Palms	Arecaceae	*Normanbya normanbyi*	59	10	23	4	14
		*Archontophoenix alexandrae*	7	2	22	0	3

One sample is equal to one microhabitat sampled on one individual tree at one point in time.

To examine patterns in species diversity between each microhabitat, all adult beetles (Coleoptera) were pinned or pointed and sorted to morphospecies (hereafter referred to as species). Species were compared with previous collections from the site [15] and were critically evaluated by CWW, NES and PSG. The beetle fauna was chosen because of its ecological diversity and high species richness [46], which allowed us to make the comparisons necessary to test our hypotheses. Microhabitat specialisation was calculated for each beetle species using *Sm* (Specificity to microhabitat *m*, analogous to the Host Specialization (*HS*) measure of Novotny et al. [47] which is based on an earlier measure by Thomas [48]). *Sm* for each beetle species is simply the proportion of the total number of individuals that were collected from the preferred microhabitat (i.e., that which supported the greatest number of individuals). *Sm* accounts for variation in beetle abundance on different microhabitats, and reduces bias caused by increasing numbers of rare records that inevitably accumulate from large sample sizes. This technique produces similar results to the commonly used Lloyd's index. Indeed, the *Sm* measure and Lloyd's index for our data were closely correlated (r = 0.98). However, Lloyd's index is a relative measure of specialisation for each species in a community, which means that it can only show that species *a* is more or less specialised than species *b*. The *Sm* method was therefore chosen as it allowed for the identification of microhabitat specificity for each beetle species (e.g., species *a* is a specialist while species *b* is a generalist).

The *Sm* method involved assigning each beetle species to one of three groups:

- Specialists: species where *Sm* >0.9.
- Preferences (or oligophages): species where 0.5< *Sm* <0.9, since most individuals (50–90%) were collected from a single microhabitat, indicating that they have a preference for it but are not necessarily specialised.
- Generalists: species where 0.33< *Sm* <0.5, since no microhabitat supported more than half of all individuals.

Assigning specialisation in this way is sensitive to absolute numbers of records per species. Specialisation analyses were therefore restricted to the 77 beetle species where at least 12 individuals were collected. The limit of 12 individuals was chosen as a compromise between including a maximum number of species and reducing errors arising from potential assignation of specialisation when none actually exists.

It should be noted that mature leaf biomass constitutes >90% of the combined biomass of the focal microhabitats (C Wardhaugh et al. unpublished data), so a randomly distributed beetle species will be assigned as a “mature leaf specialist” since >90% of its population should be found on mature leaves. It is therefore not possible to discern mature leaf specialists from randomly distributed microhabitat generalists, since both should be found predominantly on mature foliage. However, as we are primarily concerned with describing the distributions of beetles, distinguishing between specialists and generalists on mature leaves is a moot point. Therefore, for the sake of clarity, we refer to all beetles where *Sm* >0.9 on mature leaves as specialists. This is not the case for flowers and new leaves, however. The spatially and temporally restricted distribution of flowers and new leaves means random distribution of individuals across microhabitats should produce (on average) less than 10% of all records for each species on these resources. Defining microhabitat specialisation using cut-off values of >90% and >50% as employed by the *Sm* method is therefore considered robust in determining specialisation or preference for flower and new leaf beetles.

Sorensen Index (*So*) was used to measure the similarity of the beetle community within and between each microhabitat across host tree species. The *So* coefficient is a pair-wise comparison that quantifies the proportion of beetle species common to two samples. *So* ranges from 0, where there is no species overlap between host tree species, to 1, where each beetle species is distributed across all tree species. To produce a mean measure of species overlap for each microhabitat, *So* coefficients within each microhabitat were averaged across all pair-wise comparisons of host tree species. The Chao 1 biodiversity indicator was used to estimate the number of beetle species that utilise each microhabitat on the tree species studied. Sorensen coefficients and Chao 1 biodiversity indicators were calculated using EstimateS 8.20 [49].

### Microhabitat Biomass Estimation

Different microhabitats vary considerably in size and biomass both between tree species and within individual trees. As such, a time-based measure of collecting effort, where it is assumed that an equal amount of each microhabitat will be sampled during a set time period, is inappropriate to estimate invertebrate density as a function of biomass available. Furthermore, an attempt to sample an equal amount (weight, surface area or volume) of each microhabitat on each tree was unfeasible, due to the large differences in biomass between microhabitats. Therefore, we combined our time-based sampling protocol (each microhabitat was sampled on each tree for ten minutes), with an estimate of the biomass of each microhabitat in each sample to produce densities of invertebrates/kg or resource.

To calculate the biomass of a unit of microhabitat (i.e., a single leaf or flower), mature leaves and flowers were collected from each plant species, dried at 60°C for 48 hours and weighed. Mature leaves (n = 9–40/species, mean 30.7) and flowers (n = 1–10, mean 8.2) were weighed and the mean used in subsequent calculations of biomass. New leaves were distinguished from mature leaves on the basis of colour and texture. Many new leaves on a flushing tree are still expanding, and will therefore weigh much less than fully expanded new foliage. Nevertheless, measurement of all new leaves is logistically impossible. Samples of fully expanded, but not yet toughened, new leaves weighed just 56.5% (±6.7%) of conspecific mature leaves. We therefore estimated the biomass of a single new leaf to be 50% of the biomass of a conspecific mature leaf.

The amount (kg dry weight) of each microhabitat present on each tree at the time of sampling was calculated by visually estimating the number of units (leaves, flowers) of each microhabitat within tree crowns [50]. Specifically, the number of resource units (i.e., leaves, flowers/inflorescences) within five, randomly located, 1 m^3^ samples of tree crown were counted, and extrapolated to the total estimated volume (m^3^) of tree crown sampled [50]. Estimating the volume sampled was made easier by sampling in increments of 1 m^3^ of microhabitat-bearing tree crown at a time. For instances where there were few flowers or new leaves on a tree, all microhabitat units were counted rather than estimated using extrapolation. All counts and estimates were carried out by CWW to reduce any bias between different observers [50]. The estimated number of resource units sampled was then multiplied by the measured biomass of that particular resource unit to generate an estimated amount (kg) of microhabitat sampled. This provided a basis for a calculation of the density of invertebrates and beetles per kilogram of resource within each tree species, making between- and within-microhabitat comparisons possible. Densities on each microhabitat were weighted for biomass/tree species each month, to avoid potential bias produced by high densities or high microhabitat biomass on single tree species. Differences in mean density among microhabitats were examined using ANOVA.

## Results

**Figure 1:**
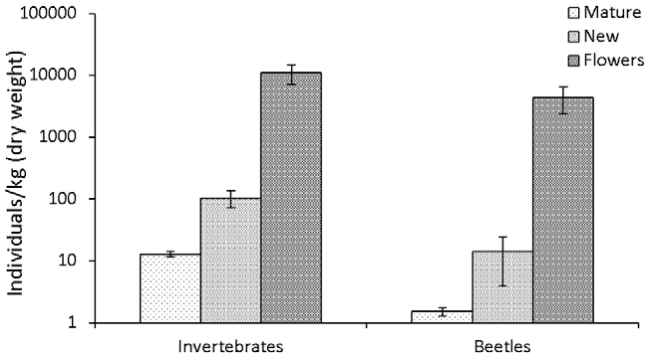
Invertebrate and beetle density on each microhabitat. The density of invertebrates and beetles on mature leaves, new leaves and flowers (per kg dry weight ± 2SE).

Over one year a total of 39,276 invertebrates, including 10,185 beetles from 358 species, were collected from mature leaves (363 trees sampled), new leaves (78) and flowers (82). Expressed per unit biomass, a disproportionately large number of individuals were associated with new leaves and especially flowers, where invertebrate densities were 1–4 orders of magnitude greater than on the foliage; a pattern consistent across all 18 canopy plant species that flowered during the study (Figure S1). The density of invertebrates per unit biomass of resource varied significantly between microhabitats (*F*_2, 56_ = 216.51, *P*<0.0001), with flowers supporting 11,055.9±1,884.3 (weighted mean ±1 SE) individuals per kilogram, and 105.0±16.4/kg on new leaves compared to just 12.8±0.7/kg on mature foliage (Figure 1). Similar differences in density were also found among the beetle fauna (*F*_2, 56_ = 181.27, *P*<0.0001), with flowers supporting 4,440.3±1,020.1 individuals/kg, compared to 14.0±5.0/kg on new leaves and 1.5±0.1/kg on mature leaves (Figure 1).

**Figure 2:**
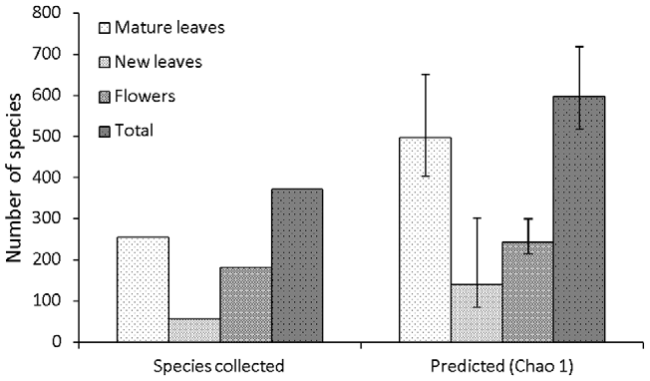
Beetle species richness on each microhabitat. The total number of beetle species collected, and the estimated number of beetle species (Chao 1 (±95% CI) species richness estimator) utilising each microhabitat. Note that the Chao 1 calculations estimate the total number of species that utilise each microhabitat, including microhabitat generalists and rare microhabitat records, and are not restricted to the number of specialist species.

**Figure 3:**
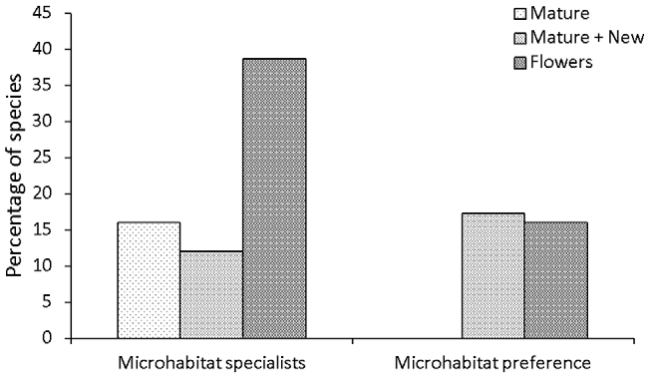
The percentage of species that are microhabitat specialists on each microhabitat. The percentage of the 75 most abundant beetle species (n≥12) that are specialised to each microhabitat (*Sm* >0.9) or showed a distributional preference for a microhabitat (0.5< *Sm* <0.9). Note that no species was specialised to new leaves, but some were specialised, or preferred, foliage in general (mature leaves and new leaves combined, identified as foliage specialists and foliage preferences).

Species level analysis of the beetle community showed a disproportionately high concentration of species on flowers. The majority of the estimated number of beetle species are expected to utilise mature leaves, reflecting the large proportion of canopy biomass this microhabitat constitutes (Figure 2). However, the Chao 1 biodiversity indicator showed that 41% of beetle species utilise flowers and 23% utilise new leaves (Figure 2), percentages far greater than the relative contributions of these microhabitats to total canopy biomass. It should be noted though, that species accumulation curves for each microhabitat did not reach asymptotes [40], which could reduce the reliability of the Chao 1 calculations. Flowers were utilised by a relatively specialised fauna, with 39% of the 77 most common beetle species collected identified as specialists (*Sm* >0.9) on this resource, compared to just 16% on mature leaves (Figure 3).

There was a greater overlap in species composition of the beetle assemblages on different host trees within each microhabitat than between microhabitats. Within microhabitats, the mean Sorensen coefficient (*So* ± SE) of the flower-visiting (0.2±0.011) and mature leaf-visiting (0.2±0.007) beetle communities was identical (*t*_382_ = 0.25, *P* = 0.8). Overlap in species composition was much lower between microhabitats, with a mean Sorensen coefficient of 0.11 (±0.004) for pairwise comparisons between the beetle communities identified from flowers and mature leaves. The mean Sorensen coefficient of the new leaf-visiting beetle community was low (0.11±0.012), possibly reflecting the lower total number of beetles collected from this microhabitat and the subsequent reduction in species overlap between different host plant species. Consequently, there was little overlap between the flower-visiting and new leaf beetle communities (*So*  = 0.04±0.003) or the mature leaf and new leaf beetles (*So*  = 0.09±0.004). No beetle species was identified as being specialised to new leaves. Rather, the new leaf beetle community was mostly a subset of the mature leaf beetle community, with 44/56 (78.6%) species collected from new leaves also collected from mature leaves. In contrast, only 88/182 (48.4%) flower-visiting species were also recorded from mature leaves.

## Discussion

Flowers represent important resources for rainforest canopy invertebrates and our data clearly demonstrate that they are sites of very high concentrations of individuals and species. We show that despite constituting a tiny fraction of the biomass of mature foliage, flowers, and to a lesser extent new leaves, harbour a large proportion of the abundance and diversity of canopy invertebrates. We also show that the assemblages associated with different microhabitats are significantly different, with flowers supporting a complementary fauna to that on leaves. Although most canopy species are expected to utilise mature foliage, the very large biomass of leaves, coupled with the very low density of beetles on this microhabitat mean that sampling the leaves only will result in a large proportion of these species going underrepresented or uncollected. As a result, the null hypothesis that invertebrate abundance and species richness is proportional to microhabitat biomass is rejected, and the hypothesis that each microhabitat is inhabited by its own relatively discrete invertebrate community is supported by our data. We can therefore also reject the assumption that the foliage-inhabiting invertebrate community can be used as a proxy for communities inhabiting other canopy microhabitats. We suggest that insects associated with flowers may be a neglected component of invertebrate diversity.

High concentrations of invertebrates on flowers may occur due to pollination rewards, floral herbivory, or because flowers act as aggregation sites for mate finding and/or because flowers attract prey for predatory species [26], [33]–[38]. We suggest that one of the reasons why invertebrates are so hyper-abundant and diverse on flowers compared to leaves could be linked to the contrasting roles that these structures serve to the tree. Leaves are long-term photosynthetic structures whose loss impacts the growth, survival and/or reproduction of the parent tree [51], [52], whereas flowers function to attract insect pollinators by providing food rewards in the form of highly nutritious and often easily digestible pollen and/or nectar [53]. Although widespread comparative analyses of the chemical profiles of flowers and foliage are lacking [36], it is not unreasonable to assume that flowers are generally nutritionally superior to leaves for most herbivores [54], since plants need to attract insect consumers to carry out pollination ([26], but see [55]). In fact, pollen-feeding is common among basal herbivorous beetle lineages and may have served as a nutritional and mechanical stepping stone towards folivory [56]. Leaves in contrast, do not benefit from herbivores and are therefore protected structurally and chemically from insect attack, which renders them nutritionally poor.

In one of the few comparative studies, Carisey and Bauce [57] showed that balsam fir (*Abies balsamea*) pollen contained lower concentrations of defensive compounds and higher levels of available nitrogen than either new or mature foliage. Indeed, it is unlikely that chemical defences should evolve to deter insect visitors from flowers, since reduction in insect floral attendants could have a detrimental impact on reproduction (but see [58], [59]). For example, *Brassica rapa* plant populations in Montana display variability in concentrations of the enzyme myrosinase. Potential pollinators spend more time foraging in populations with low myrosinase concentrations compared to populations in which flowers express high concentrations of this enzyme, indicating that defensive compounds in floral tissues can negatively effect pollination [60].

A number of studies have shown that the tough structure of leaves is an effective herbivore defence [3]. However, the ephemeral nature of flowers results in less structural defences such as lignified cell walls and fibre [61] compared to long lasting leaves. Insects that consume the lignified cell walls of leaves must typically consume large quantities of this material and pass the undigested cellulose in the excreta, even though it can constitute a high proportion of their food intake [62]. Flowers may therefore represent concentrations of high quality accessible food surrounded by lower quality and largely inedible foliage, resulting in spatially aggregated concentrations of diverse invertebrate consumers.

Several lines of evidence suggest that flowers are likely to support a similarly high proportion of the canopy insect community in other rainforests. First, other studies have also found that flower-and foliage-associated invertebrates represent distinct assemblages in both rainforests [25] and in other biomes [21]. Second, 20 of the 23 plant species sampled in our study come from families that are pantropical in distribution; Arecaceae, Myristicaceae, Lauraceae, Proteaceae, Euphorbiaceae, Fabaceae, Myrtaceae, Sterculiaceae, Meliaceae, Apocynaceae, and Convolvulaceae. The remaining two families, Elaeocarpaceae and Cunonaceae, are also distributed beyond Australia. Third, beetle communities inhabiting rainforest canopies are remarkably similar across the tropics in terms of the rank order of families in species richness [7], [63]. Fourth, beetles are relatively conservative in their feeding biology at the family/subfamily level [64]. All of these factors reduce the likelihood that the result we report is a local phenomenon driven by host tree phylogeny or beetle assemblage composition, and suggest that our findings may be indicative of tropical rainforests in general.

Our data show that species overlap between host plants among the flower-visiting beetle assemblage was relatively low. This suggests that flowers from individual host plant species harbour a diverse and relatively specialised community at the local scale. We also found similar levels of dissimilarity in beetle species composition on mature foliage across tree species. In contrast, Ødegaard [24] found that flower-visiting herbivorous beetles were less specialised than folivorous beetles. However, that study was restricted to beetles from three herbivorous superfamilies (Buprestoidea, Chrysomeloidea, and Curculionoidea), and did not assess other prominent and diverse flower-visiting families such as Staphylinidae, Nitidulidae, and Phalacridae [65], [66]. Species from these latter families were common in our flower samples and displayed a similar level of host specialisation compared to members of the herbivorous superfamilies examined by Ødegaard ([24], [25], C Wardhaugh et al. unpublished data).

Our findings of high host specificity of flower-visiting species in a tropical rainforest have important implications. If, as seems most possible, our results are applicable to rainforest systems in other locations, the increase in estimated host specificity could lead to increased global biodiversity estimates [6]–[11]. This will depend on two pieces of information. These are empirical data on the host specificity of flower-visiting species in other tropical rainforests, and reliable estimates describing the relationship between alpha (local) and gamma (regional) diversities at larger spatial scales. Many tree species in tropical rainforests occur at very low densities [44] and are thus often isolated from flowering conspecifics. Flower-visitors may therefore need to range widely to find their desired resources [67]. If true, it is possible that flower-visiting species may be more wide-spread than foliage inhabiting species (i.e., display lower levels of beta diversity) than folivorous species, which would reduce estimates of the global biodiversity of flower-visitors. Also, since many flower-visiting species from predominantly non-herbivorous beetle families are not counted as herbivores [13], [15], [68], estimates of global species richness have typically counted them as non-herbivores using a correction factor based on Erwin's [6] original estimate [9], [14]. This correction factor assumes low levels of host specialisation as the species it is intended to cover, such as predators, do not rely on plant resources, and are thus less likely to become host plant specialists. Our data suggest that this correction factor is inappropriate when trying to account for flower-visitors since flowers attract many species from traditionally non-herbivorous groups that display relatively high levels of host specialisation (C Wardhaugh et al. unpublished data).

Our results demonstrating the concentration of insects on the small biomass of flowers has wide-ranging implications for those attempting to further our understanding of plant-herbivore interactions and canopy food webs [5]. Recent attempts to quantify rainforest food webs have ignored flower-visiting insects. Kitching [69] developed a simple rainforest food web in an attempt to identify components/linkages for which adequate information currently exists, and those that require further investigation. While Kitching's [69] model incorporated plant, herbivore, predator/parasitoid, and detritivore diversity, the flower-visiting component was not addressed. Similarly, in one of the most comprehensive examinations of a rainforest food web to date, Novotny *et* *al.* [5] examined the trophic links between 224 plant species and 1,490 species of herbivores from 11 distinct feeding guilds. Leaf feeders, xylem and phloem feeders, fruit feeders, and gall formers were studied, but flower-feeders were omitted from their analyses due to a lack of data. Spatial and temporal aggregations of very high densities of flower-visiting invertebrates could result in a high number of strong interactions, making flowers an ideal habitat to study intra- and interspecific interactions among a species-rich community. Flower-visiting invertebrate food webs, where resource availability and the resulting invertebrate abundances may fluctuate widely, are therefore likely to be more dynamic than those based on more widely available and reliable resources such as the leaves. Furthermore, since flowers and their components lack many of the defences typical of leaves, species from non-herbivorous feeding guilds often feed on floral resources. For example, many parasitoid wasps and flies consume nectar [68], blurring the line between herbivore and predator.

If flower-visiting insects do indeed represent a larger component of rainforest biodiversity than previously thought, then we need to re-evaluate our current theories and estimates relating to the spatial and temporal distribution of insects in rainforest canopies. The exclusion of flowers from diversity studies in tropical rainforests could previously be justified by canopy access issues and the small biomass of flowers compared to the foliage. Furthermore, those studying herbivory generally dismiss flower-visitors as pollinators [26], while pollination biologists typically focus on the few species in the community that carry out successful pollination [33]. The result has been the omission of many herbivores and an entire community from food web analyses and species richness estimates [26]. But, as we have shown, abundance and diversity estimates that do not include flower-visitors, or are derived from sampling the foliage-inhabiting community alone are unlikely to be indicative of the entire canopy fauna. Substantial microhabitat partitioning among arboreal invertebrate communities means that sampling mature leaves misses a large number of species altogether. The potential for the flower-visiting fauna to contribute significantly to global biodiversity and food web dynamics emphasises the need to account for this assemblage in future studies of rainforest biodiversity.

## Supporting Information

Figure S1 **a–v.** The density of invertebrates on each microhabitat on each tree species. The density/kg (± S.E.) of invertebrates on each of the 22 tree species for which at least two of the three focal microhabitats were sampled (all species except *Musgravia heterophylla*). Note that the data are presented on a log scale. Missing columns signify that no samples from that microhabitat on that plant species were taken.
(TIF)
